# Resilience in advanced cancer caregiving promoted by an intimate partner’s support network: insights through the lens of complexity science. A framework analysis

**DOI:** 10.1186/s12904-023-01134-3

**Published:** 2023-02-17

**Authors:** Sophie Opsomer, Sofie Joossens, Emelien Lauwerier, Jan De Lepeleire, Peter Pype

**Affiliations:** 1 Academic Centre for General Practice, Department of Public Health and Primary Care, KU Leuven, Kapucijnenvoer 33, box 7001, 3000 Leuven, Belgium; 2 Department of Public Health and Primary Care, Ghent University, Ghent, Belgium; 3 Program of Health, University Colleges Leuven - Limburg, Leuven, Belgium; 4 Department of Experimental Clinical and Health Psychology, Ghent University, Ghent, Belgium; 5 End-of-Life Care Research Group, Ghent University Campus, Ghent, Belgium

**Keywords:** Complex adaptive system, Complexity science, Resilience, Advanced cancer, Caregiver, Support network, Palliative care

## Abstract

**Background:**

The tremendous physical and mental burden that comes with caregiving puts the intimate partners of patients diagnosed with advanced cancer at risk for mental disorders. However, most partners seem to be protected by resilience. Such a resilience process is promoted by certain individual characteristics (e.g., flexibility, positive attitude, internal strength, capacity to balance incoming and outgoing information, and ability to ask for and accept support and advice) and by the availability of a support network, consisting of family, friends, and healthcare professionals. Such a heterogeneous group striving towards the same goals can be considered a complex adaptive system (CAS), a concept stemming from complexity science.

**Aims:**

To study the behavior of the support network through the lens of complexity science and to provide insights to the means by which an available network may promote resilience.

**Methods:**

Nineteen interviews with members from the support networks of eight intimate partners were analyzed deductively using the CAS principles as a coding framework. Subsequently, the quotes under each principle were coded inductively to concretize patterns in the behavior of the support networks. Eventually, the codes were charted into a matrix to identify intra- and inter-CAS similarities, differences, and patterns.

**Findings:**

The network’s behavior adapts dynamically to the changing circumstances as the patient’s prognosis worsens. Furthermore, the behavior is based on internalized basic rules (such as reassuring availability and maintaining communication without being intrusive), attractors (e.g., feeling meaningful, appreciated, or connected), and the history of the support network. However, the interactions are non-linear and often unpredictable due to the context member’s own concerns, needs, or emotions.

**Conclusions:**

Applying the lens of complexity science to the behavior of an intimate partner’s support network gives us insight into the network’s behavioral patterns. Indeed, a support network is a dynamic system that behaves according to the principles of a CAS and adapts resiliently to the changing circumstances as the patient’s prognosis worsens. Moreover, the behavior of the support network appears to promote the intimate partner’s resilience process throughout the patient’s care period.

**Supplementary Information:**

The online version contains supplementary material available at 10.1186/s12904-023-01134-3.

## Background

The extraordinary mental and physical burdens imposed upon the intimate partner (the person with whom one has an intimate relationship) of a patient diagnosed with advanced cancer places the intimate partner at risk for depression, anxiety, or even post-traumatic stress disorder [1, 2]. Yet most intimate partners seem to resist the psychological strain that comes with caregiving and cope adaptively, protected against mental distress by resilience [2–5].

A partner being diagnosed with advanced cancer can be considered a potentially traumatic event (PTE), and the diagnosis is often followed by a period of intense emotions [5]. However, a PTE usually initiates a resilience process, defined by the American Psychological Association (APA) as “the process and outcome of successfully adapting to difficult or challenging life experiences, especially through mental, emotional, and behavioral flexibility and adjustment to external and internal demands” [6]. Yet how well one adapts to a PTE, such as a partner being diagnosed with advanced cancer, is influenced by one’s baseline adjustment, referring to how one functioned and adapted to other challenges prior to the diagnosis [7]. Furthermore, a resilience process is promoted by some individual characteristic traits such as flexibility, a positive attitude, the capacity to keep incoming and outgoing information in balance, being able to ask for and accept support and advice, and internal strength [8]. Moreover, coping strategies such as focusing on daily life, taking up responsibility, and managing or mastering the situation, are known as moderators of the resilience process [8]. Additionally, resilience is influenced by the availability of a support network [8, 9]. Indeed, from a recent meta-synthesis of studies on resilience in cancer caregiving, it can be concluded that most caregivers feel reinforced when surrounded by people who care about them and with whom they can share their concerns [8]. Such a support network, composed of family, friends, and professional caregivers, can be considered a complex adaptive system (CAS): a network consisting of a heterogeneous group of individuals who act autonomously although not always in a predictable way [10, 11]. Furthermore, the individual actions are interconnected, meaning that one group member’s action will change the context for other group members and will provoke a reaction. Hence, in a CAS the interactions are more important than the individual actions of the group members [10, 11]. As such, complexity-based reasoning suggests that resources should be allocated to the entire group rather than to the individuals. Consequently, difficult goals and solutions to complex problems can be attained by a CAS in which behavior follows a few simple principles – where the CAS: has fuzzy boundaries, acts according to internalized basic rules, is characterized by non-linear and attractor-based behavior, is prone to tension, is adaptive, and in which the behavior is based on the CAS’ history [10, 12]. An overview of the general CAS principles, applied to a network of people, is given in Table 1.

**Table 1:** Key principles of complexity science as applied to social systems behaving as complex adaptive systems (CAS)

**Principle**	**Meaning**
Fuzzy boundaries	The system is open. Each member of the system is embedded in other systems. Although one member responds almost exclusively to its primary member, the dynamic interactions within adjacent systems affect the interactions within the index system
Internalized basic rules	Each member of the system acts autonomously, driven by instincts and constructs, which has been learned by previous experiences
Non-linear behavior	Small variations in input can cause very significant changes in output. Even when members interact only with a few others, the effects are propagated throughout the system
Attractor-based behavior	Rewarding interactions will produce repeated behavior either immediatelly or after a series of intervening stages. This may result in increased integrity, autonomy, and ideals
History-based, path-dependent behavior	Systems evolve. The past is partially responsible for present behavior. Systems are sensitive to their initial conditions. Hence, the same force might affect systems differently dependent on initial conditions
Unpredictability, tension, and paradox	The overall behavior of the system is not predicted by the behavior of the indvidual elements. The system oscillates between order and chaos. A constant flow of energy is needed to maintain the organization of the system
Adaptivity	The system’s internal structure is (re)organized without external intervention. The interactions are more important than individual actions. The interactions are interconnected and lead to novel behavior. Systems that are simultaneously ordered and disordered are more resilient

Analyzing the behavior of the networks supporting the intimate partner of a person diagnosed with advanced cancer overall and through the lens of complexity science may provide novel insights not yet identified from traditional descriptive studies or other relevant frameworks, such as studies with a socio-psychological approach. Indeed, social psychology has a long history of research in caregiving, focusing on how the behavior, motivation, and thoughts, of the individual caregivers are influenced by their context [13]. However, to fully understand the behavior of a support network, a complementary stance, starting from the behavior of the network as a whole is fundamental. Furthermore, insights into the behavior of a support network may shed light on how this behavior could be related to resilience.

Therefore, this study aims to answer the following research question: How are the principles of a CAS expressed in the behavior of a network supporting an intimate partner of a patient diagnosed with cancer in an advanced stage?

## Methodology

The reporting of this study is based upon the Consolidated Criteria for Reporting Qualitative Research (COREQ criteria) [14]. The 32-item COREQ checklist along with its corresponding pages is provided as an additional file. [See Additional file 1].

### Research team and reflexivity

This interview study was conducted by a multidisciplinary research team consisting of researchers experienced in palliative care, primary care, complexity science, psychology, and qualitative research. The first author, a family physician experienced in palliative care and in qualitative research, initiated the study and conducted the interviews as part of her PhD project. Prior to commencement of the study, no professional nor personal relationship was established between the interviewer and the participants.

### Study design

#### Theoretical framework

A thematic framework analysis of the data, stemming from semi-structured interviews, was undertaken [15, 16]. Through the development of a matrix, framework analysis allows for combining themes-based and case-based analyses and for identifying patterns across cases [16, 17].

#### Participant selection

The target population of this study is the network of family, friends, and professional caregivers supporting the intimate partner of a patient diagnosed with advanced cancer – defined as a patient diagnosed with cancer in stage III or IV or with metastatic cancer. Eight intimate partners – all participants in an ongoing longitudinal qualitative study on resilience in cancer caregiving – were informed verbally about the study objectives and design. An additional file provides more details about the in- and exclusion criteria of the longitudinal study [See Additional file 2]. During the interview for the aforementioned longitudinal study, each of the intimate partners was requested to invite three or four people to participate in the present study. The candidates were eligible to participate (1) if they were considered indispensable according to the patient’s intimate partner (because of the mental or practical support they offered to this partner while caring for a patient with advanced cancer), and (2) if they were fluent in Dutch. Nineteen potential candidates contacted the researcher by email or phone. They received extensive written information regarding the study. After signing a written informed consent, the researcher contacted them to make concrete arrangements for the interview.

#### Setting

The interviews were conducted between April 2020 and May 2021. To limit personal contacts during the COVID-19 pandemic, seventeen interviews took place via Zoom and were video recorded. The other two interviews were conducted in the interviewer’s office with a wall of plexiglass between the interviewer and the interviewee. These two interviews were audio recorded. The interviews lasted between 40 and 75 min, were transcribed verbatim, and were not translated into English. After completion of the analysis, the recordings of the interviews were deleted from the interviewer’s computer.

**Fig. 1:**
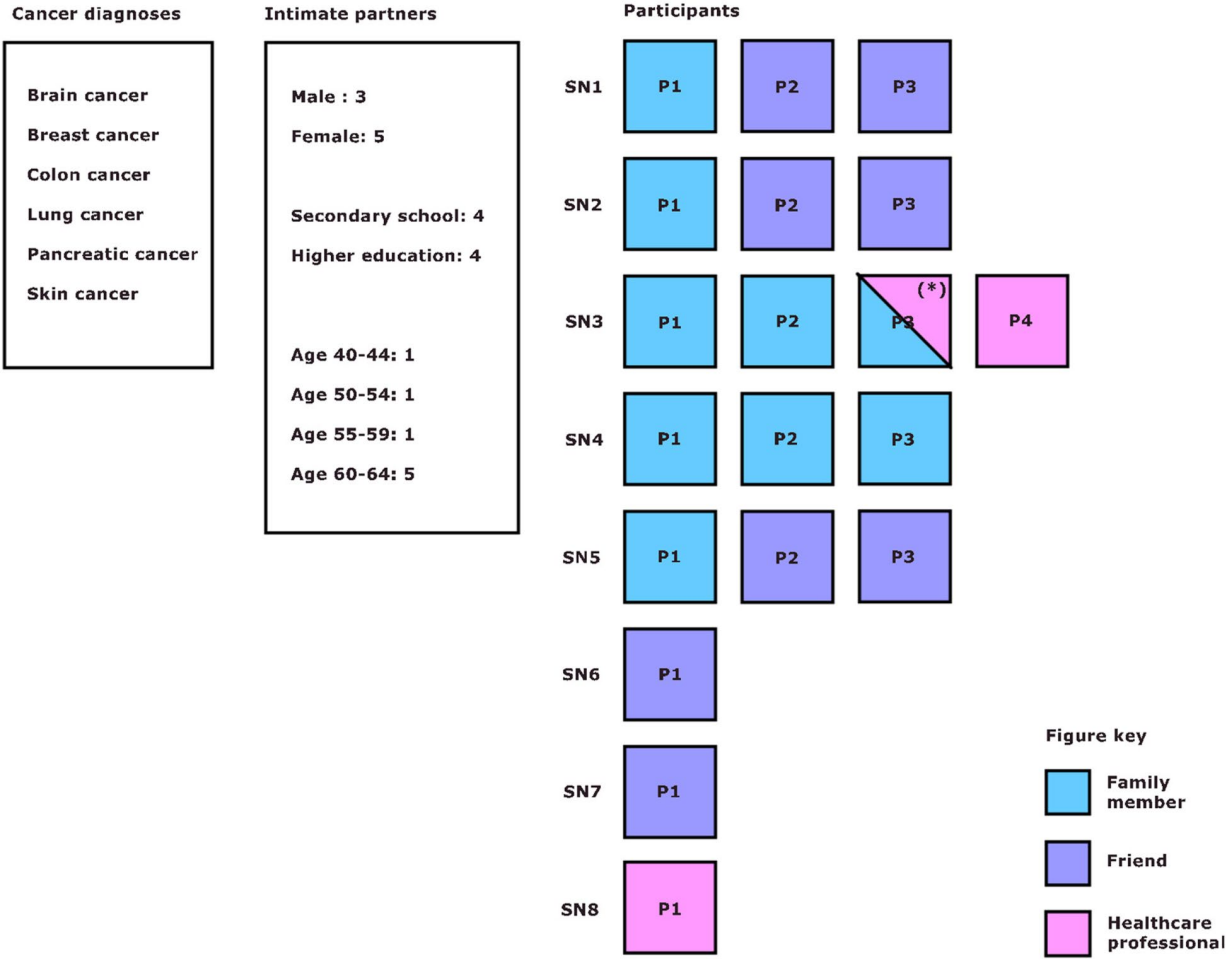
SN: support network. P: participant. (*) One professional caregiver was also a family member

Figure 1 provides more details about the patients, their intimate partners, and the composition of their support networks.

#### Data collection

The interview guide was designed and based on literature on complex adaptive systems [18] and resilience [8] and comprised the following topics: 1. Experiences in being part of a meaningful network of an intimate partner of a patient with advanced cancer; 2. Assumed tasks and roles; 3. Communication within the supporting network; 4. Reasons to continue the support. Field notes were made to ensure reflexivity. The translated interview guide is provided as an additional file. [See Additional file 3].

### Data analysis

**Fig. 2:**
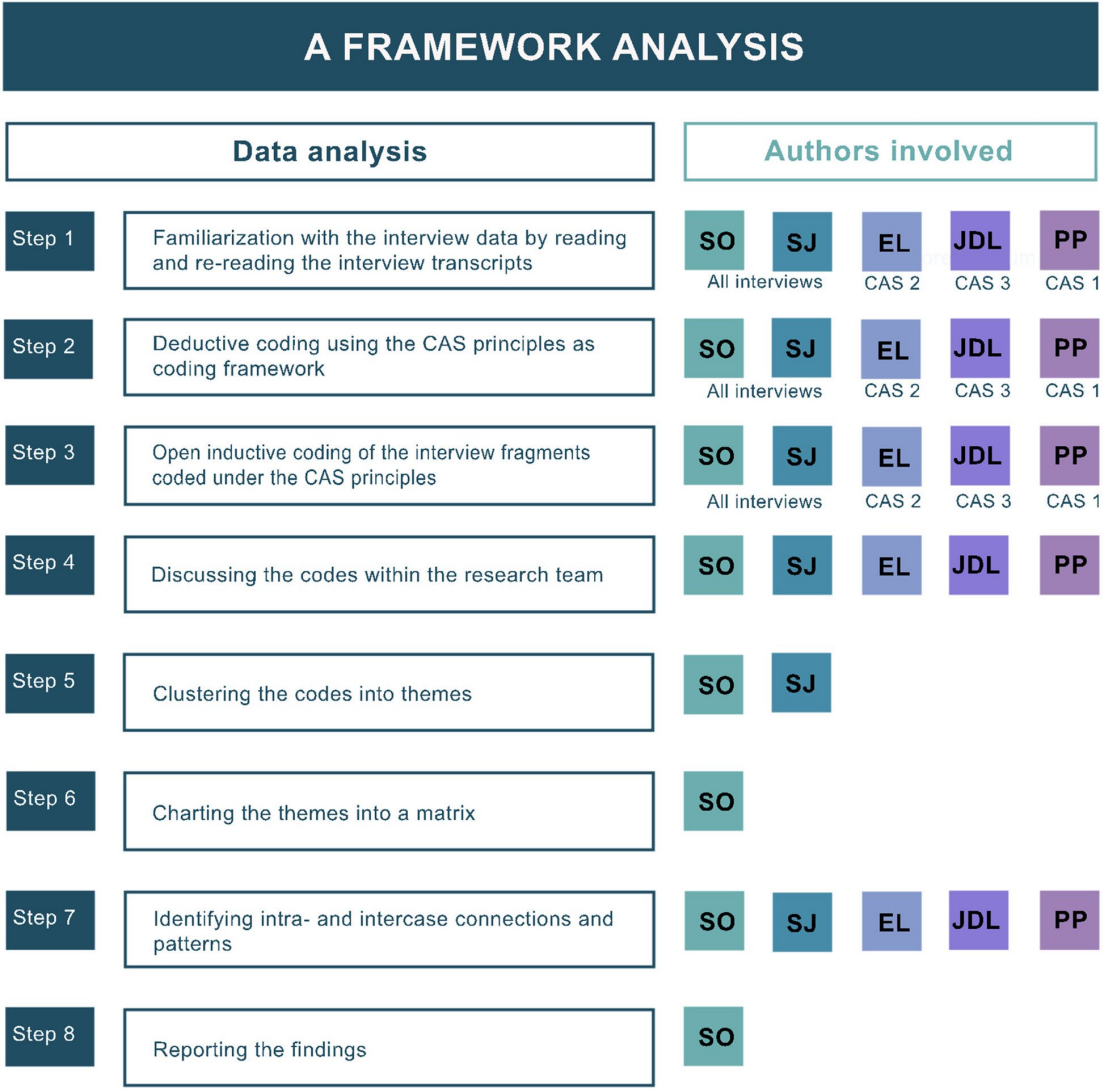
In eight steps, 19 interviews, resulting from 8 CASs, were analyzed deductively and inductively. CASs 1–3: steps 1–3 (3, 3, and 4 interviews respectively) were carried out by 3 researchers independently. CASs 4–8: steps 1–3 (3, 3, 1, 1 and 1 interviews respectively) were carried out by 2 researchers independently. All interviews: steps 4 and 7 were carried out by the entire research team. All interviews: step 5 was carried out by 2 researchers

In a first step, the CAS principles, described above in Table 1, were used as a framework for deductive coding of the data. Consequently, the interview fragments coded under each CAS principle, were further analyzed inductively and sorted into themes. The coding of all interviews was performed by two researchers independently (SO and SJ) and discussed until agreement on all codes and themes was reached. To ensure trustworthiness, reliability, and credibility of the findings, the coding of the interviews belonging to the first three networks was checked and commented on by one of the other team members (PP, EL, JDL, respectively). Language support was provided by a native English-speaking American instructor to properly distinguish nuances in the coding. Therefore, some representative quotes were translated into English. During the next phase, and according to the framework method, the codes (in English) and their illustrating quotes (in Dutch) were charted in a matrix where the CAS principles are placed in columns and the supporting networks (the CASs) in rows. Finally, the matrix was discussed by all researchers involved to identify intra- and inter-CAS similarities, differences, and patterns. Details on the authors’ contributions to the analysis are given in Fig. 2.

### Ethics

The study conforms to the declaration of Helsinki. Ethical approval was provided by the Ethics Committee Research UZ / KU Leuven on October 4, 2019, study number S63166 and by the Ethics Committee of Ghent University Hospital on October 17, 2019, study number BC-06066.

## Findings

Nineteen participants, being part of a CAS of an intimate partner of a patient with advanced cancer, provided a rich account on how they perceived being part of one of the eight studied support networks. The resultant matrix from the analysis of their stories allowed for all CAS principles to be identified in most of the studied support networks surrounding the intimate partners. However, the principles were adopted in variant ways, resulting in a range of behavioral patterns, as described below. Obviously, in support networks represented by only one participant, some CAS principles were not discussed. For instance, the fuzziness of the CAS boundaries remained unclear, and the participants did not report on any tension or paradox within the support network. The matrix resulting from the analysis is given as Table 2. In the illustrating quotes participants are represented as SN (support network) 1–8 and P (participant) 1–4.

**Table 2:** Matrix illustrating how the CAS principles are represented in the support networks’ behavior

CAS principle		Fuzzy boundaries	Internalized basic rules	Non-linear interactions	Attractor-based behavior	History-based behavior	Tension and paradox	Adaptation
Support network 1	Family 1 Friends 2 Healthcare professional (HP): 0	A1 – A2	B1 – B2	C1	D1 – D3	E1	F1 – F3	G1 – G3
Support network 2	Family 1 Friends 2 HP: 0	A1	B2 – B4 – B5	C2	D1 – D2 – D3	E1 – E2	F1 – F2 – F3	G1 – G2
Support network 3	Family 3 Friends 0 HP: 2	A1 – A2	B1 – B2 – B4 – B5	C2 – C3	D1 – D3	E2	F3	G1 – G2 – G3
Support network 4	Family 3 Friends 0 HP: 0		B1 – B2 – B3 – B4 – B5	C1 – C2	D1 – D2 – D3	E2		G1 – G2 – G3 – G4
Support network 5	Family 1 Friends 2 HP: 0	A1	B1 – B2 – B4	C1 – C2	D1 – D3	E2	F1	G2
Support network 6	Family 0 Friends 1 HP: 0	A2	B2 – B3	C1 – C4	D1	E1 – E2	F1	G1
Support network 7	Family 0 Friends 1 HP: 0		B1 – B2	C1 – C3	D3	E1 – E2		G2
Support network 8	Family 0 Friends 0 HP: 1		B2	C4	D1			G2 – G3

A1: Being aware of other groups in the partner's support network

A2: Sharing emotions and concerns beyond the group's boundaries

B1: Maintaining communication without being intrusive

B2: Reassuring availability of guidance and support with respect for the autonomy of the intimate partner

B3: Acknowledging the intimate partner's emotional vulnerability in an empathic way

B4: Providing reciprocal support and assistance to loved ones

B5: Avoiding being an extra burden to the intimate partner

C1: Reassuring availability and support for autonomy can elicit non-linear behavior

C2: Discussing the future, exhibiting gestures of goodwill, or sharing experiences can evoke non-linear emotional reactions

C3: Explaining the intimate caregiver's situation can elicit non-linear empathic reactions

C4: COVID-19 measures can lead to overly cautious behavior

D1: Feeling meaningful and appreciated

D2: Recognizing sources of joy and spreading positivity throughout the support network

D3: Feeling connected and enjoying each other’s company

E1: Sharing more experiences and forging closer relationships mean assistance is more easily offered and accepted

E2: Making an effort to maintain continuity in meaningful relationships

F1: Reassuring availability for guidance and support is hampered by the context member's own concerns and needs

F2: Empathic involvement can be hindered by the need to cope with one's own emotions

F3: Setting aside pre-existing personal history and issues

G1: Resulting from a worsening prognosis, the communication style adapts to this new reality

G2: Contextualizing the internalized basic rules

G3: Strengthening the feeling of togetherness and maximizing intimate group contact as the prognosis worsens

G4: Allowing for the natural evolution of roles within the system

### Fuzzy boundaries

Each member of a CAS is incorporated in other CASs. Although one member responds almost exclusively to the person with whom they are in direct contact, the dynamic interactions can spread throughout adjacent CASs.

#### Being aware of other groups in the intimate partner’s support network

Most participants were aware of existing networks or people involved in the support of the intimate partner. Although some could describe in detail what these networks did and how they supported the intimate partner, others had no insight into the actions of other networks.

*He does have friends over there. One of his friends is a psychologist, so he can tell him anything. (SN5 – P3)*

#### Sharing emotions and concerns beyond the group’s boundaries

The more the participant’s and the intimate partner’s lives were intertwined – either through family ties or through shared experiences – the more they shared their emotions with each other. The participants admitted that they could not always cope with their emotions prompted by their commitment on their own. Hence, they shared their stories with other people who were less involved or who did not take part in the intimate partner’s support network.

*My friends know about it [the patient’s story], and occasionally we talk about it. If I tell it to one friend and I say: this is terrible … Of course, it’s terrible. But if it doesn’t happen in your own household, fortunately it doesn’t affect you as much. (SN6 – P1)*

### Internalized basic rules

Although each member of a support network acts autonomously, internalized basic rules shape their behavior.

#### Maintaining communication without being intrusive

The participants expressed their commitment and willingness to listen to the intimate partner’s stories and they created opportunities to talk. For instance, they invited the intimate partner for a walk, to have tea in the garden, or to sit on the front porch. Here, they respected the intimate partners by leaving the initiative to talk with them without asking questions themselves. Furthermore, they strived toward an open and honest communication by not avoiding difficult topics.

*But most of all, she felt the need to talk about it [how she experienced her husband’s diagnosis]. And, I thought, let’s get her out of her house. Let’s have a cup of coffee together and talk. Or we could go for a walk so that she feels comfortable to tell her story. I wanted her to be alone with me so that she could talk freely without her husband around.* (SN1 – P2)

#### Reassuring availability of guidance and support with respect for the autonomy of the intimate caregiver

Professional caregivers, family, and friends expressed their unconditional availability for guidance and support of the intimate partner. However, they did not intervene and waited patiently for the intimate partner to take the initiative.

*Then we told her: “Mom, if you think we could do something to help you, just ask. Don’t feel embarrassed. Yeah, we have our own life but dad and you, you’re so much more important than our job or anything else.”* (SN4 – P1)

#### Acknowledging the intimate caregiver's emotional vulnerability in an empathic way

The participants felt most appreciated by the intimate partner when empathy was expressed. Moreover, acknowledging the intimate partner’s vulnerability could positively influence the relationship between the partners and their support networks.

*If something would go wrong with her [the patient], I’m almost 100% sure he [the intimate partner] will break. I try to avoid this by talking to him regularly. Not to lecture him, but to listen and to say: “Yes, if you did everything in your power…” I’ve certainly made mistakes myself which I’ll regret for the rest of my life. But, I’m just a human being, right? With my gifts and faults. But I try to support him. It won’t be easy for him. It’s already difficult, that’s for sure.* (SN6-P1)

#### Providing reciprocal support and assistance to loved ones

The participants emphasized how they unconditionally supported and assisted the intimate partner, often driven by connectedness, a strong sense of reciprocity, or a genuine affection for their family member or friend.

*I would say, well, she’s my mom. I love her very much. She was always there for me too. What she’s going through now is probably the most difficult thing she’ll have to endure in her life. So, the least I can do is to be there for her.* (SN3-P1)

#### Avoiding being an extra burden to the intimate partner

To allow the intimate partner to focus on the care of the patient only, the participants illustrated how they avoided being a burden to the intimate partner themselves by hiding their emotions of grief and sadness and by attending to their own self-care.

*How can I get rid of this blanket of depressive feelings that’s hanging over me? It doesn’t help me and [when I have negative feelings myself] I won’t be able to care for someone else either, right?* (SN4-P1)

### Non-linear interactions

The behavior of a CAS is characterized by non-linear interactions that can be positive (a modest action causing a disproportionate reaction) or negative (an action eliciting a minor or no reaction).

#### Reassuring availability and support for autonomy can elicit non-linear behavior

When the support network members adhered to the internalized basic rule of reassuring availability with respect for the unconditional autonomy of the intimate partner, they often remained passive, waiting for the intimate partner to take the initiative. However, the same internalized basic rule could elicit positive non-linear behavior such as responding to a request without delay or adjusting travel plans in order to maintain a line of unbroken communication and a quick return home should the situation warrant.

*By being attentive and responding to his queries, right? If he needs me, I’m there as quickly as 112 [emergency number in Belgium], that’s for sure. (SN6-P1)*

#### Discussing the future, exhibiting gestures of goodwill, or sharing experiences can evoke non-linear emotional reactions

Being informed about the patient’s diagnosis of advanced cancer could remind the support context members of former experiences that consequently elicited excessive emotional reactions, expressing themselves in various ways, such as crying or displaying avoidance behavior.

*When [the patient] was diagnosed, the housemaid said: “Oh, my brother also died because of cancer and I’m afraid to see that phlegm again. I can’t deal with this anymore.” After this she said that she didn’t want to come anymore. Since then, she doesn’t visit my mom any longer either. So, it has become an awkward situation, and it was another hit mentally for my mom. (SN3-P1)*

In addition, intense emotions could be provoked in the intimate partner (e.g., by discussing the future or by providing them with a gift). Such gestures confronted the partners with their own difficult situation and the contrast to all those not facing a health crisis.

*I’d bought her [the intimate partner] flowers. “You spent your money on* this*?” she asked. A small flower when I felt she wasn’t coping well. So, yeah, I brought her flowers, beautiful flowers, the smallest bouquet [laughs]. I know she likes receiving flowers. But at first, she didn’t want to accept the bouquet. However, at night, she started sending messages to say: “I’m sorry for being so brutal. All those people around me seem to be happy, and I must always pretend [to be happy as well].” After messages like these, I knew she was not doing well.* (SN2-P2)

#### Explaining the intimate caregiver's situation can elicit non-linear empathic reactions

In certain cases, intimate partners waited to share their stories and explain the circumstances in which they found themselves. After disclosure though, they discovered that people began to offer empathy and expressed a willingness to support.

*At work, she [the intimate partner] pinned a leaflet to the wall stating that her husband was palliative. After, you could see that a lot of customers were suddenly startled and much friendlier towards her. People that are otherwise very strict and rigid now showed empathy and became involved. It all feels strange but I think that’s very comforting to her. (SN3-P1)*

#### COVID-19 measures can lead to overly cautious behavior

All interviews were conducted during the COVID-19 pandemic. The fear of infecting the patient and the measures in force at that time resulted in extremely careful behavior and avoiding all physical contact.

### Attractor based behavior

In a CAS, attractors shape the behavior of the system. Accordingly, recurring actions of the studied support networks could be framed as a result of their striving toward the following three attractors:

#### Feeling meaningful and appreciated

Family members and friends as well as professional caregivers illustrated how gratefulness and appreciation sharpened their intrinsic motivation to support the intimate partner. For instance, they attended to the others’ needs, shared meaningful experiences, and did their best to create memorable moments.

*I never feel forced to do anything. I do all this of my own will. I’m the daughter-in-law now [hesitates] and I want to be a good one. I want to be there for those people, even in bad times. They are my family now. And above all, I know that I’m also doing my boyfriend a favor. But a simple thank you is already enough. It feels good when you can do something meaningful for them.* (SN5-P1)

#### Recognizing sources of joy and spreading positivity throughout the support network

The participants were attentive to what events could generate happiness and joy. As such, they intended to spread positivity throughout the intimate partner’s support network as often as possible.

*I know that they [the patient and partner] love their granddaughters. That’s their source of joy. The more they see them, the better. That’s what makes a person happy. Just seeing them walk around or being able to talk to them. I know that’s important. And me, well, we are not the kind of people who take a hundred pictures or videos of their children, but we deliberately share these with them more often now.* (SN4-P2)

#### Feeling connected and enjoying each other’s company

The intimate partner’s friends and family shared how they strived to meet as often as possible (e.g., by regularly visiting, inviting the intimate partner for a walk), simply because they enjoyed being together or because they felt strongly connected.

*We also often said to each other: “Shall we go and see how the grass is growing?” [laughs]. So, we sat down on a bench, drank something and talked about all kinds of things, including the cancer and [the patient] and about him [the intimate partner]. He liked this and it was nice for me too.* (SN5-P2)

### History-based behavior

The history and experiences CAS members share and their mutual relationships can stimulate the dynamic interactions within a CAS as well as paralyze them.

#### Sharing more experiences and forging closer relationships means assistance is more easily offered and accepted

As a result of closer relationships and shared experiences between the intimate partner and support context members, an increase in assistance was offered and accepted. Moreover, the intimate partner, by accepting help, motivated the support context to put forth more suggestions for help, thereby often overriding the internalized basic rule of leaving the initiative to the intimate partner.

*I’ve never pushed him to talk because I didn’t know him well enough. But now, I would try to convince him a bit sooner, since now we get along very well. But back then, I was more cautious. In the past, I would have left him alone and if we weren’t going to talk, it was okay.* (SN5 – P1)

#### Making an effort to maintain continuity in meaningful relationships

When meaningful relationships within the support network threatened to break, the members made efforts to restore them by encouraging contact between each other or by reminding others of the responsibilities assumed in a relationship.

*It was the same with my brother. She [the intimate partner] said: “I haven’t heard from him in a week, that’s not normal.” And yes, that isn’t normal because in that week [the patient’s] health deteriorated dramatically. So, I sent my brother a message that said: “Look, you really should call mom because she needs you, you can’t let her down.”* (SN3-P1)

### Tension and paradox

When tension arises between the internalized basic rules and one’s own concerns or emotions, the behavior of the CAS becomes increasingly unpredictable, even paradoxical.

#### Reassuring availability for guidance and support is hampered by the context member's own concerns and needs

Although the intimate partner’s family and friends emphasized the importance of being available for guidance and support whenever necessary, they sometimes preferred to take care of their own concerns and needs first.

*[Two friends were shopping and having coffee together when the intimate partner called them]. She insisted we both come over [to talk]. We both wondered if we should go see her or not. However, we decided not to go since it was our day off and that we both work full time, and since she [the patient] was in hospital and was being well cared for.* (SN2-P2)

Moreover, a participant pointed out that providing guidance and support was actually an internalized basic rule prone to non-linearity since the condition was ultimately relinquished to the intimate partner.

*If I can support him [the intimate partner]… Well, I tell you this in confidence that, in fact, no one in the world can help him. There’s only one person who can solve that problem [dealing with the patient’s cancer diagnosis] and it’s the caregiver himself. You can hand him a tool, but if he doesn’t know how to use it, he can’t do anything with it. In the end, everybody should be a bit self-taught.* (SN6-P1)

#### Empathic involvement can be hindered by the need to cope with one's own emotions

Providing emotional support was easier when the members of the support network were not hindered by their own feelings or were less emotionally involved (e.g., when they were not connected to the patient or if they actually lived far away). Furthermore, emotional interactions could be disrupted when one had to deal with opposite feelings stemming from different roles in an adjacent CAS, as one friend pointed out how difficult it was to support the intimate partner who was grieving since she had recently fallen in love and felt the happiest on earth.

*I want to be there for her [the intimate partner], but her partner [the patient] is not really my friend. I like her, and she’s always welcome here, that’s not the point. But I decided not to get involved too much. I thought I should be there for her [the intimate partner] in the first place. If she breaks down, I don’t want to have to deal with my own grief at the same time.* (SN2-P2)

#### Setting aside pre-existing personal history and issues

Despite bad relations with the intimate partner, some family members unconditionally adhered to the internalized basic rule: one should be available for one’s family under any circumstance. For them, the quality of the relationship was subordinate to the need of being available.

*From the start, I just flipped the switch in my head and said to myself: “I must be there for her. I’ll put myself second for now. I really must be there for her. I should try to help her wherever I can so that her life will be a bit easier again.” And that’s exactly what I’ve done.* (SN1-P1)

### Adaptivity

During the caregiving process for a patient with advanced cancer, the behavior, actions, and communication of the intimate partner’s support context evolve and adapt resiliently to the specific needs related to the cancer stage.

#### Resulting from a worsening prognosis, the communication style adapts to this new reality

Shortly after a patient was diagnosed with advanced cancer, the communication within the support network and with the intimate partner was mostly spontaneous and open. Discussions about difficult topics such as death or dying were encouraged and old irritations were put aside. However, when the patient’s prognosis worsened, the communication within the support context often became more structured, more deliberate, and less spontaneous. One support network even established an information circuit to guarantee communication under all circumstances.

*I just know that we, my siblings and I, noticed that it all was too much for mom, with the administration, the care, and so on. So, at a certain moment, we decided to have an island council as we called it, a family council. It wasn’t my mother’s idea, but one of my sisters who arranged this.* (SN4-P1)

#### Contextualizing the internalized basic rules

The internalized basic rules were reversed when the situation became too demanding for the intimate caregiver. For example, when the context members realized that the partner was getting overloaded, instead of adopting a wait-and-see attitude, they set aside the basic rule (reassuring availability of guidance and support with respect for the autonomy of the intimate caregiver) and intervened by making any necessary decisions or by taking over tasks.

*And at that moment, the doctor said: “Someone has to come now [to help with the care for the patient].” So, the GP basically decided for her that it was too much right now and that she couldn’t do this all alone anymore.* (SN3-P3)

#### Strengthening the feeling of togetherness and maximizing intimate group contact as the prognosis worsens

The support network matched the care supply to the demands of the intimate partner. As such, when the patient’s prognosis worsened, the number of contacts increased due to an increased sense of togetherness, and the support network members often took up a shared responsibility to support the intimate partner.

*I stayed there once during the night. Well, in his last moments, she was never alone with him. Her daughter was there too, and I never thought it would be possible for me to stay with him until the very end, but, yeah, it felt so natural and it all happened spontaneously. I think it’s most important that he could stay and die at home, surrounded by [his loved ones] in his living room, and never alone. For her [the intimate partner] too, that must have been most comforting.* (SN3-P2)

#### Allowing for the natural evolution of roles within the system

In the support networks, each member took on a role at their own discretion. However, in time, the networks became more organized as people took up different roles.

*If I describe the team, my mother [the intimate partner] is the leader, the project manager and we are the team members. We all have different roles in this. There is [one of the siblings], who’s always the prepared reader and is the one who provides information in a way we can all understand and make use of it. [Another sibling] mirrors my mom and tends to be an emotional buffer. I’m the ice breaker. If things are left unspoken, I initiate the discussion*. (SN4-P1)

## Discussion

Based on the complexity science framework, this study reveals new insights into how members of a network supporting an intimate partner of a patient diagnosed with advanced cancer interact.

Their behavior is based on internalized basic rules (such as reassuring availability and maintaining communication without being intrusive), attractors (e.g., feeling meaningful, appreciated, or connected), and the history of the support network. However, the interactions are non-linear and often unpredictable due to the context member’s own concerns, needs, or emotions. Nevertheless, the network’s behavior adapts dynamically to the changing circumstances as the patient’s prognosis worsens.

Pype et al. (2018) [18] and Hodiamont et al. (2019) [11] studied the professional palliative healthcare teams and professional palliative care situation respectively, according to the CAS principles. They described how patterns of interactions structure the functioning of the professional teams and shape the dynamics of palliative care structures. Our study contributes to how networks – composed of family, friends, and healthcare professionals supporting the patient’s intimate partner – likewise behave as a CAS. Moreover, we can state that the same CAS principles as applied to the professional setting by Pype et al. [18], can be applied to the support networks around the intimate partner, albeit with an adapted concrete implementation.

Since the support network is self-organizing, adapts to the challenges of a worsening prognosis, and adjusts to the demands and needs of the intimate partner, it is apparent that the members’ behavior applies to the dynamics of a resilience process defined as the process of adapting well to adversity [19]. However, being supported by a resilient network as such cannot guarantee the emergence of a resilience process in the individual. Nevertheless, from earlier studies on resilience in cancer caregiving, it is well-known that the availability of a support network is paramount in the development of the intimate partner’s resilience process [5, 8, 20]. Indeed, our findings underpin how the behavior of the support context can refine the intimate partner’s characteristics needed to enable a resilience process. For instance, the internalized basic rule of reassuring availability for guidance and support with respect for the intimate partner’s autonomy could enhance adaptive dependency, an important resilience promoting characteristic trait, which demonstrates that the intimate partner is more eager to ask for and to accept help. Additionally, the context member expressing willingness to listen without being intrusive, should make it easier for the intimate partner to maintain control over the information flow. Moreover, as the support context members set aside negative thoughts and emotions and instead shared as many beautiful moments as possible, positive feelings tended to spread throughout the support network. Finally, respecting the intimate partner’s autonomy and acknowledging their needs, may also enhance their inner strength [8].

The interactions within the support network could even indirectly facilitate more of a resilience process by consolidating the intimate partner’s sense of coherence [21]. As such, comprehensibility, meaningfulness, as well as manageability of the caregiving situation may be strengthened by the support context by maintaining communication while respecting the intimate partner’s space, acknowledging their vulnerability, reassuring availability and unconditional support, and by maintaining continuity in meaningful relationships [21]. Consequently, although the general behavior of the support context might be unpredictable, one could indeed be reassured that the support would most likely adapt to the circumstances as well as can be expected.

### Strengths and limitations of the study

To our knowledge, the current study is the first to systematically explore the behavior and interactions within the support network of a partner of a patient diagnosed with advanced cancer through the lens of complexity science. Complexity science focuses on the adaptability of a CAS, oscillating between order and chaos, while taking into account its unpredictability. As a result, this approach seemed most suitable to study patterns and interactions of the behavior of a support network that functions in a rapidly changing context characterized by unpredictability [10]. A noteworthy strength of the study is the varied composition of the studied support networks, represented by family, friends, as well as professional healthcare providers. The heterogeneity of the support networks also allowed for diverse perspectives. Moreover, analyzing the data by a Framework Method required frequent, extensive and critical discussions on how the quotes from the interviews were related to the pre-existing CAS principles. This interdisciplinary team approach may occasion a rigorous qualitative analysis enhancing the relevance and credibility of the findings.

This study was also subject to limitations. All interviewees were of Flemish descent, and the majority (17/19) were highly educated, having attained a university or university college degree. Consequently, our findings apply only to a well-defined group and cannot simply be transferred to other groups, such as people from immigrant origin or people living in non-European countries. In addition, one must be critical when applying the findings to support networks composed of family and friends who are less educated. Finally, all interviews were conducted during the COVID-19 pandemic. The fear of infecting the patient and the measures that were taken by the government unmistakably influenced the intimate partner’s resilience process [22]. As a result, the partners were often reluctant to have contact with others or to allow people to enter their houses. Hence, an ideal adaptive process of the support network could have been hampered and several interactions might have been hindered as spontaneous meetings were mostly avoided.

### Implications for practice and research

By studying the behavior of the networks supporting an intimate partner of a patient diagnosed with advanced cancer through the lens of complexity science, patterns in actions and reactions could be framed within the behavior of a CAS in accordance with the universally applicable CAS principles. The new insights stemming from this study will allow healthcare professionals to understand the dynamics of a support network in which they often participate themselves. Healthcare professionals should allow the support network to evolve and adapt as a system rather than focusing on the individual actions, including their own. Moreover, it is important to recognize the network’s emerging internalized basic rules as they emphasize the autonomy of the intimate partners as a holistic focus – in communication as well as in emotional and practical support. As such, allowing for a CAS to establish will promote a person-centered (i.e., intimate partner-centered) approach [23]. Furthermore, endorsing the attractors might be an efficient way to motivate the network members to persevere and to maintain positivity. However, more research among diverse population groups is necessary in order to expand our findings.

## Conclusion

Family, friends, and healthcare professionals can form a support network around the intimate partner of a patient diagnosed with advanced cancer. Once a support network is established, one can expect it to behave in accordance to the CAS principles. As such, the system acts in agreement to its internalized basic rules, driven by attractors. Although the behavior is non-linear and not fully predictable, the system is dynamic and resilient and adapts to the changing circumstances as the patient’s prognosis worsens. Finally, the behavior of the support network according to the principles of a CAS, appears to promote the intimate partner’s resilience process throughout the care period of the patient.

## Supplementary Information

**Additional file 1. **COREQ (COnsolidated criteria for REporting Qualitative research) Checklist.pdf The 32-items COREQ checklist for interviews and focus groups, along with the corresponding pages.

**Additional file 2. **Inclusion and exclusion criteria – longitudinal study on resilience in advanced cancer caregiving.pdf Details on the in- and exclusion criteria of a longitudinal study on resilience in advanced cancer caregiving. Participants (intimate partners) in the longitudinal study recruited the participants (members of the support networks) of the current study.

**Additional file 3. **Interview guide.pdf A translated version (from Dutch to English) of the interview guide on which the semi-structured interviews were based.

## Data Availability

The datasets used and analyzed during the current study are available from the corresponding author on reasonable request and with permission of the Ethics Committee Research UZ / KU Leuven. The interview transcripts will be delivered in the original language (Dutch) and will be anonymized.

## Abbreviations

APA: American Psychological Association
CAS: Complex adaptive system
HP: Healthcare professional
PTE: Potentially traumatic event
