# Supplementary material for: Resilience in advanced cancer caregiving promoted by an intimate partner’s support network: insights through the lens of complexity science. A framework analysis
Source: BMC Palliat Care. 2023 Feb 17;22:12. doi: 10.1186/s12904-023-01134-3 (PMC9936125; doi:10.1186/s12904-023-01134-3)
Supplement: Supplementary file 2 — Additional file 2. Inclusion and exclusion criteria – longitudinal study on resilience in advanced cancer caregiving.pdf Details on the in- and exclusion criteria of a longitudinal study on resilience in advanced cancer caregiving. Participants (intimate partners) in the longitudinal study recruited the participants (members of the support networks) of the current study. [file 12904_2023_1134_MOESM2_ESM.pdf]

Additional file 2

Inclusion and exclusion criteria – longitudinal study on resilience in advanced cancer caregiving.

Details on the in- and exclusion criteria of a longitudinal study on resilience in advanced cancer caregiving. Participants (intimate partners) in the longitudinal study recruited the participants (members of the support networks) of the current study.

Inclusion criteria

- Being the partner and principal caregiver of a person recently (less than six months) diagnosed with cancer in an advanced or palliative stage. Advanced stage cancer is defined as cancer in stage III, IV, or metastatic cancer. Cancer in a palliative stage means that the goal of a cure is no longer reasonable or life expectancy is one year or less.
- Adults under 65 years of age.
- Fluency in Dutch.

Exclusion criteria

- Partners with diagnosed depression or psychological illness before the cancer diagnosis.
- Partners of patients with a life expectancy of three months or less.
