# Supplementary material for: Resilience in advanced cancer caregiving promoted by an intimate partner’s support network: insights through the lens of complexity science. A framework analysis
Source: BMC Palliat Care. 2023 Feb 17;22:12. doi: 10.1186/s12904-023-01134-3 (PMC9936125; doi:10.1186/s12904-023-01134-3)
Supplement: Supplementary file 3 — Additional file 3. Interview guide.pdf A translated version (from Dutch to English) of the interview guide on which the semi-structured interviews were based. [file 12904_2023_1134_MOESM3_ESM.pdf]

Additional file 3

Interview guide

A translated version (from Dutch to English) of the interview guide on which the semi-structured interviews were based.

- By whom and how were you informed about X's condition (X = the patient)?
- Can you tell us what your first reaction was when you learned the news about X?
- Did you immediately see a task for yourself in the support of X's partner Y?
    - How did you fill that role?
    - Were others involved?
    - Can you tell me more about this?
- Did you talk to anyone about supporting Y outside the so-called team?
    - Have you sought advice from anyone?
    - Did anyone give you unsolicited advice?
    - In what ways did you find out what is expected of you and how you should act upon that?
- How would you describe the support of Y?
- Has anything changed in your relationship with Y since the diagnosis?
    - How would you describe this?
    - What triggered this change?
- Do you have contact with other family members/friends/caregivers of X or Y? We will refer to this group as the team from now on.
    - Did you also know these people before the diagnosis?
    - Has anything changed in your relationship with Y? What has changed?
- Can you give a brief overview of who is on the team? Who is involved in caring for Y or X and what is each person's role?
- Can you describe how communication within the team works?
    - Who takes the initiative?
    - What is and is not communicated?
    - With whom do you communicate and with whom do you not?
- When do you struggle with this care? When does it feel more like a task?
    - Can you clarify this with an example?

- How do you deal with this?

- When do you feel like everything is working out?

  - Can you provide an anecdote of this?

- Looking at the whole network or team around Y, how would you situate yourself in this? How do you yourself function within this group?

- If you could dismiss one person from the group, who would it be and why?

- If you were allowed to choose one person who should definitely stay in the group, who would this be and why?

- What makes you continue to provide this care?

- Under what circumstances would you stop providing care?
